# Genetically predicted vitamins supplementation and risk of skin cancers: a Mendelian randomization study

**DOI:** 10.1007/s12672-025-01905-9

**Published:** 2025-02-19

**Authors:** Ping Qi, Liyun Chen, Aiwei Ma, Yiwen Zhang, Hai Lin, Wenqi Shi, Zijian Huang, Zixuan Tang, Wenshi Jiang, Mengjing Xu, Wancong Zhang, Shijie Tang

**Affiliations:** 1 Department of Plastic Surgery and Burns Center, Second Affiliated Hospital, Shantou University Medical College, Shantou, 515051 Guangdong China; 2 Plastic Surgery Institute of Shantou University Medical College, Shantou, 515051 Guangdong China; 3 Shantou Plastic Surgery Clinical Research Center, Shantou, 515051 Guangdong China; 4 Research Center of Translational Medicine, Second Affiliated Hospital of Shantou University Medical College, Shantou, 515051 Guangdong China

**Keywords:** Vitamin, Folate supplementation, Mendelian randomization, Skin cancers, Meta-analysis, MRlap

## Abstract

**Background:**

The causal relationship between vitamins supplementation and the risk of skin cancers remains unclear. We conducted a Mendelian randomization study to assess the associations between vitamins supplementation and skin cancers in the general population.

**Objective:**

We aimed to investigate the causal relationship of vitamins (A-E) supplementation with skin cancers using and utilizing the linkage disequilibrium score regression (LDSC) Mendelian randomization (MR).

**Materials and methods:**

We selected genome-wide significant single-nucleotide polymorphisms (SNPs) linked to vitamin supplements (A-E). Summary-level data for melanoma, basal cell carcinoma, and squamous cell carcinoma were obtained from large-scale genome-wide association studies. We applied the inverse-variance weighted (IVW) method within a random effects model, alongside weighted median, MR-Egger, simple median, sensitivity analyses, and MRlap methods to ensure robustness. A meta-analysis was conducted to assess the causal relationship between vitamins supplementation and melanoma. The STROBE-MR checklist was followed throughout.

**Results:**

Folate supplementation was associated with a reduced melanoma risk (IVW OR = 0.88; 95% CI 0.80–0.96; P = 0.006). The MR analysis indicated a significant inverse causal relationship. Heterogeneity and sensitivity analyses confirmed minimal impact from individual SNPs. MRlap corrected for potential estimation bias due to sample overlap, which was not significant, reinforcing the IVW findings. The meta-analysis ensured robust and stable results. LDSC regression analysis suggests a weak causal relationship between folate supplementation and melanoma. Yet no association was found between genetically predicted vitamins A, B6, B12, C, D, E, and skin cancers.

**Conclusion:**

Both observational meta-analysis and MR analysis based on genetic variation provide robust evidence indicat that folate supplementation decreases the risk of melanoma, suggesting that interventions targeting folate supplementation may contribute to the primary prevention of melanoma. Further studies are needed to explore the potential association between other vitamins supplementation and the risk of skin cancers.

**Supplementary Information:**

The online version contains supplementary material available at 10.1007/s12672-025-01905-9.

## Introduction

Skin cancers, one of the most commonly occurring cancers worldwide, are generally categorized as melanoma, basal cell carcinoma (BCC), and squamous cell carcinoma (SCC) as the major subtypes and constitute the major proportion of all skin cancers cases [1, 2]. However, compared to the high incidence and low mortality rate of SCC and BCC, cutaneous melanoma (CM) is known to cause significantly more deaths [3]. Melanoma is a common skin cancer globally, with a significant presence in the form of cutaneous melanoma (CM), which arises from melanocytes in the basal layer of the epidermis in the skin [4]. This malignancy occurs when the melanocytes responsible for producing pigment in the skin undergo a malignant transformation in the epidermis [5]. According to a recent global survey [6], current forecasts propose that by 2040, there will be a significant increase in the number of melanoma cases, reaching up to 510, 000 new cases (a roughly 50% increase) and 96, 000 deaths (a 68% increase). These statistics highlight the urgent need for effective prevention, early detection, and supplementation measures, as well as improved access to healthcare services for those at high risk of developing skin cancers.

Retinoids are natural and synthetic derivatives of vitamin A that are effective for preventing and treating non-melanoma skin cancers (NMSC) [7]. It's a new concept that vitamin may contribute to preventing skin cancers [8]. Vitamin B repairs UV-induced DNA damage reduces UV-related skin immunosuppression, and lowers the risk of certain skin cancers [9]. Vitamin B also reduces transepidermal water loss and the development of new non-melanoma skin cancers in high-risk individuals [10]. Vitamin C (VitC) is known to impair cancer cell growth directly in preclinical models, but there is little clinical evidence of its antitumoral efficacy [11]. However, high doses of vitamin C have significantly reduced cancer cell viability and invasiveness, and induced apoptosis in human malignant melanoma [12]. The current vitamin D deficiency epidemic is accompanied by an increase in endemic skin cancers [13]. Other research suggests a potentially causal positive association between serum 25-Hydroxyvitamin D levels and melanoma risk, challenging traditional beliefs about vitamin D's role in melanoma [14]. As for Vitamin E, it is the major naturally occurring lipid-soluble non-enzymatic antioxidant that protects the skin from the adverse effects of oxidative stress, including photoaging [15]. The role of vitamin E in maintaining normal bodily functions and its effectiveness in treating skin cancers has been widely debated [16].

Folate, (vitamin B9), vitamin B6 and vitamin B12 are essential water-soluble vitamins that play a crucial role in the maintenance of one-carbon metabolism [17]. Some studies have suggested a protective role of increased folate supplementation against melanoma [18], while others suggest no significant association or produce contradictory results [19]. The discrepancies in the epidemiological evidence regarding folate and melanoma incidence are often attributed to confounding variables such as lifestyle factors. Folate deficiency is an independent risk factor for cancer and folate supplementation has the potential to decrease the risk of cancers depending on the dosage, timing of folate supplementation, and type of cancer [20]. More research gradually found that folate could be used as a protective factor against tumors. Kim et al. demonstrated that 6 months of folic acid supplementation resulted in higher colonic folate levels and fewer strand breaks in the tumor suppressor gene *p53* as well as an increased extent of global DNA methylation as compared to a placebo group [21]. Lower levels of vitamin B (12) were found in patients with laryngeal squamous cell cancer [22] and melanoma [23], while Competition studies with excess native B12 resulted in a 95% decrease in tumor accumulation [24]. Vitamin B6 metabolism is required for CD8 + T cell proliferation and effector differentiation [25]. In another research supraphysiological doses of vitamin B6 can inhibit the growth of melanoma cells both in vitro and in vivo [26]. Therefore, the association of folate, vitamin B12, and vitamin B6 with skin cancers is still debated.

The potential causal relationship between vitamins supplementation and three types of skin cancers (CM, BCC, and SCC) has not been studied using MR (Mendelian Randomization) to date. To investigate this, we used a two-sample Mendelian Randomization approach [27] and Linkage Disequilibrium Score Regression (LDSC) to determine the genetic correlation from GWAS (Genome-wide association studies) summary statistics. We combined association estimates using a meta-analysis of a common effect model. The MR methodology uses Single Nucleotide Polymorphisms (SNPs) as instrumental variables (IVs) to estimate causal relationships, helping to mitigate bias from confounding factors and reverse causation, which are common challenges in observational studies [27–29]. It relies on assumptions crucial for ensuring the validity and reliability of causal inferences drawn from MR analyses [30]. Large-scale, publicly available GWAS datasets were used for SNP and outcome data.

## Materials and methods

### Study design

Our study used LDSC and a classic two-sample Mendelian randomization model. When making clinical decisions, it's important to consider the limitations of the MR method, individual health factors, and the complex nature of melanoma progression.

**Fig. 1:**
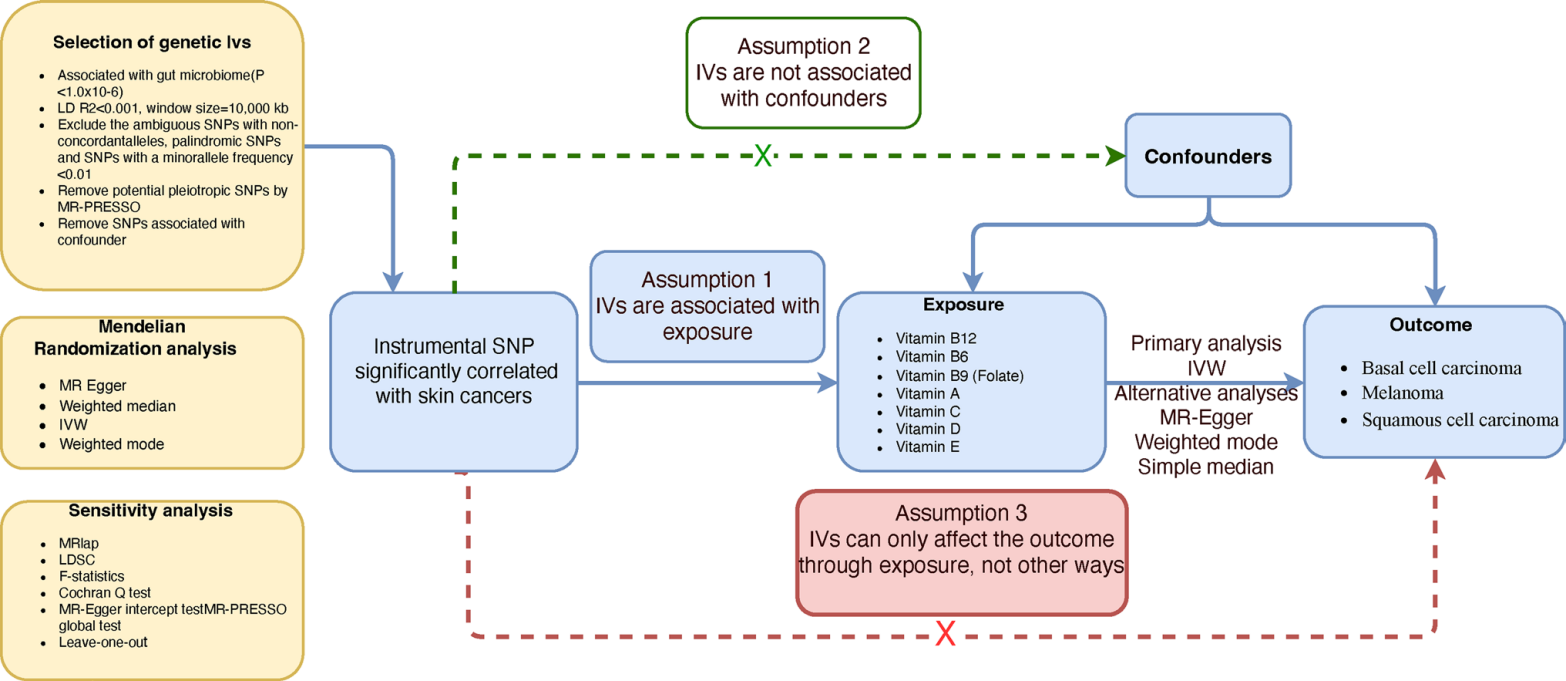
Overview of this Mendelian randomization (MR) analysis. Assumption 1, genetic instruments are strongly associated with the exposures of interest; Assumption 2, genetic instruments are independent of confounders; Assumption 3, genetic instruments are not associated with outcome and affect outcome only via exposures. IVW, inverse variance weighted; LD, linkage disequilibrium; LDSC, linkage disequilibrium score; MR-PRESSO, MR-Pleiotropy RESidual sum and outlier; SNPs, single nucleotide polymorphisms

The study is based on three key assumptions of Mendelian randomization (MR): relevance, independence, and exclusion restriction [29]. We conducted LDSC and two-sample MR to estimate the genetic correlation and causal relationships between the genetically predicted vitamins and skin cancers. An overview of the study design is presented in Fig. 1. Finally, we used the fixed-effect model to meta-analyze the MR estimates based on the GWAS of IEU and the UK Biobank.

We used the R software because it offers powerful statistical tools and can handle large genomic datasets, which are necessary for this MR study [31]. The TwoSampleMR package (4.1.0) facilitated two-sample MR analysis by offering functions for data extraction, harmonization, and conducting various statistical methods. Meta-analysis was implemented using the Metaan package. Meanwhile, the RadialMR package, which is based on modified second-order weights, enabled the identification and subsequent removal of outliers, enhancing the robustness of the analysis [32].

### Data was acquired for exposure and outcome

Genetic variants for vitamin A (ukb-b-9596), folate (ukb-b-288), vitamin B6 (ukb-b-129), vitamin B12 (ukb-b-19524), vitamin C (ukb-b-488), vitamin D (ukb-b-12648), and vitamin E (ukb-b-12506) were obtained from GWAS with European individuals. The study population involved in the GWAS was European. We identified SNPs that showed strong associations with vitamins, using a stringent threshold for statistical significance (P < 5*10^–6^), linkage disequilibrium (LD) r^2 < 0.001, and LD distance > 10,000 kb (refer to Supplementary Table 1). This meticulous selection process ensures a reduction in false-positive outcomes, thereby enhancing the reliability of our instrumental variables (IVs). Leveraging extensive and thorough GWAS datasets ensures the strength and external validity of our conclusions [33]. The stringent criteria set for SNP selection help ensure the quality of IVs and the accuracy of subsequent analyses. The F statistic was used to rule out weak instrument bias that might violate the first MR assumption and to evaluate the strength of the association between SNPs and folate supplementation levels [33, 34].

We collected summary statistics of skin cancers from published genome-wide association studies (GWASs) focusing on CM (ieu-b-4969), BCC (ukb-b-8837), and SCC (ukb-a-60) datasets with participants of European ancestry in Table 1. These datasets met the criteria for importation from the European Bioinformatics Institute (EBI) database and underwent [35] rigorous quality control checks for genotyping accuracy and consistency. Data harmonization was performed to align effect alleles and standardize measurement units for analysis. A minor allele frequency (MAF) cut-off of 0.01 was applied to ensure adequate population frequency for all included SNPs [36]. The quality, accuracy, and authenticity of each dataset were thoroughly assessed to maintain the integrity of the findings. To prevent outcome database bias, we selected 6 databases from different platforms for further meta-analysis [37]. The STROBE checklist [38] for MR studies (STROBE-MR) was employed to evaluate the quality of included studies (in supplementary STROBE-MR-checklist).

**Table 1:** Details of data sources of exposure and outcome used in Mendelian randomization

GWAS ID	Trait	Year	Cases	Controls	Population
ukb-b-9596	Vitamin A supplementation	2018	8863	451,488	European
ukb-b-129	Vitamin B6 supplementation	2018	1478	63,471	European
ukb-b-288	Vitamin B9 supplementation	2018	3883	459,050	European
ukb-b-19524	Vitamin B12 supplementation	2018	1330	63,619	European
ukb-b-488	Vitamin C supplementation	2018	5778	457,155	European
ukb-b-12648	Vitamin D supplementation	2018	17,879	442,472	European
ukb-b-12506	Vitamin E supplementation	2018	13,548	446,803	European
ieu-b-4969	Melanoma supplementation	2021	3751	372,016	European
ukb-b-8837	Basal cell carcinoma	2018	4290	458,643	European
ukb-a-60	Squamous cell carcinoma	2017	404	336,755	European

### Statistical analyses

We used four different MR methods (inverse-variance weighted, weighted median, simple median, and MR-Egger regression) to examine the causal relationship between vitamins supplementation and skin cancers. The IVW method relies on a weighted regression that minimizes errors, while the weighted median and MR-Egger methods are less powerful but useful for identifying potential biases. MR-Egger also helps check for pleiotropy, where the genetic tools might affect the outcome through other pathways besides folic acid. If pleiotropy is present and not detected, it can lead to biased and misleading results. We assessed the correlation between the selected SNPs and potential confounding factors to ensure methodological robustness and adherence to the second MR assumption, as any correlation is unacceptable. We estimated the liability-scale heritability and genetic correlation of folic acid supplementation and melanoma using LDSC [39]. Bivariate LDSC was used to estimate the genetic correlation between folic acid supplementation and melanoma. The latent causal variable model assumes that the genetic correlation between two traits is mediated by a latent variable. The genetic causality proportion indicates the extent to which the heritability of each trait is explained by a shared latent factor: higher estimates suggest a causal effect, while lower estimates suggest correlated pleiotropy. Given that our summary statistics are exclusively from European populations, the possibility of sample overlap cannot be ruled out. To mitigate potential bias from our inability to directly calculate the sample overlap rate, we used the MRlap function to adjust the IVW results. MRlap is an R-based tool for Mendelian randomization, developed to address potential sample overlap in genome-wide association studies (GWAS) [40]. Since our summary statistics are based on a European population, the risk of sample overlap is a valid concern. To mitigate this, we used MRlap to adjust the IVW results. If the corrected effects are consistent with the original estimates and show no significant differences, the IVW-MR results are reliable. If the corrected effects closely match the observed effects with no significant differences, we can trust the IVW-MR estimates. However, if there is a substantial discrepancy, the corrected effect should be prioritized, as it is independent of sample overlap. We conducted a sensitivity analysis using MRlap software, which accounts for sample overlap through an LD-score regression-related technique. We used a p-value threshold of 5 × 10 − 6, an LD threshold of 0.001, and a distance threshold of 500 kb to select instrumental variables in the MRlap analysis.

### Sensitivity analyses

The genetic causality proportion indicates the portions of heritability of each trait that are explained by a shared latent factor. Higher magnitude estimates suggest a causal effect, while lower magnitude estimates suggest correlated pleiotropy. The Cochran Q-test was used to assess the statistical heterogeneity of SNPs using IVW estimates, with a p-value of less than 0.05 indicating significant heterogeneity. Additionally, the "leave-one-out" analysis examined the reliability of individual instrument effects and assessed whether particularly influential SNPs existed [41] by systematically excluding each SNP and re-estimating the IVW effect. Furthermore, the MR-Egger method was used to determine whether the instrumental SNPs have multiple effects, and the intercept obtained from the MR-Egger regression was used to measure the directional pleiotropy [42]. Statistical significance was set at a P-value less than 2.38 × 10^–3^ (P = 0.05/(seven exposures × three outcomes) corrected for two exposures and four outcomes using the Bonferroni method [43]). A P-value above the Bonferroni-corrected threshold but lower than 0.05 was considered suggestive evidence for a potential causal association.

### Meta-analysis

Because there was a significant amount of variation between publications (P < 0.05, I2 > 50%), we used random effects models to calculate pooled effect sizes. If the variation was not significant (P > 0.05, I2 < 50%), we used fixed-effects models. We utilized Egger's test and funnel plots to evaluate publication bias. To ensure the reliability of the results, sensitivity analyses were conducted by removing each study one by one. The meta-analyses were performed using Stata 16.0 and considered statistically significant at p < 0.05. We also conducted a sensitivity analysis by removing one outcome at a time to assess the stability of the analysis.

## Results

### The causal effect of vitamins supplementation on skin cancers

**Fig. 2:**
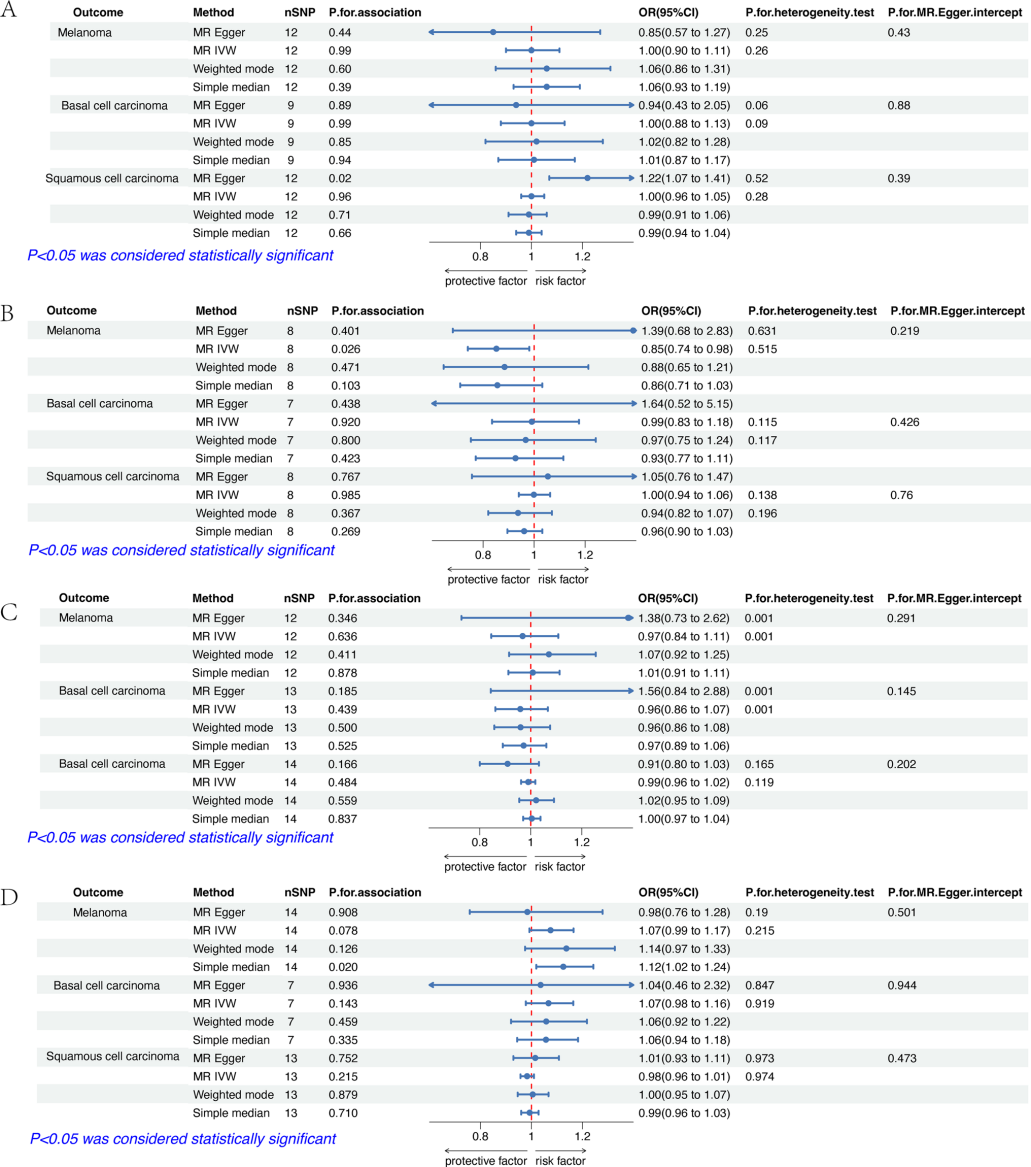
Results of two-sample Mendelian Randomization analysis assessing the risk of vitamins (**A, C, D** and **E**) on skin cancers. **A** The estimates of the causal effect of genetically predicted vitamin A supplementation on skin cancers. **B** The estimates of the causal effect of genetically predicted vitamin C supplementation on skin cancers. **C** The estimates of the causal effect of genetically predicted vitamin D supplementation on skin cancers. **D** The estimates of the causal effect of genetically predicted vitamin E supplementation on skin cancers. SNP, single nucleotide polymorphism; IVW, inverse variance weighted; MR, Mendelian randomization; OR, odds ratio; CI, confidence interval

**Fig. 3:**
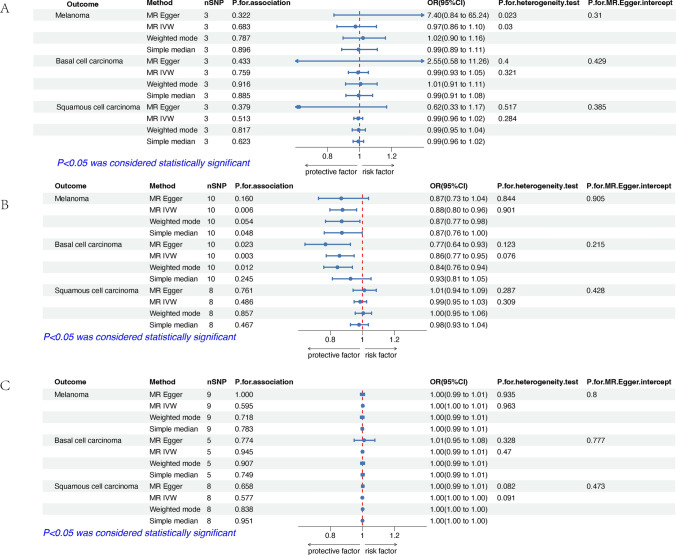
Results of two-sample Mendelian Randomization analysis assessing the risk of vitamins (B6, folate and B12) on skin cancers. **A**: The estimates of the causal effect of genetically predicted vitamin B6 supplementation on skin cancers. **B**: The estimates of the causal effect of genetically predicted Vitamin B9 (folic acid) supplementation on skin cancers. **C**: The estimates of the causal effect of genetically predicted Vitamin B12 supplementation on skin cancers

The effect estimates of the MR analyses for the association of vitamins (A, C, D, E) supplementation with skin cancers are depicted in Fig. 2. There was no evidence for statistical significance between vitamins (A, C, D, and E) supplementation with skin cancers by adopting the fixed-effect IVW model for MR analysis, or the alternative methods (supplementary Table 2). Figure 3 shows the estimates of the causal effect of genetic vitamins (B6, folate, B12) supplementation on 3 skin cancers risks. Our results demonstrated that folate supplementation was associated with lower levels of CM risk, while genetically predicted folate supplementation was not associated with other 2 skin cancers (BCC and SCC). Vitamin B6 and B12 supplementation was not associated with the risk of 3 skin cancers. Details of SNP related to folate are in Table 2. A statistically negative significant causal association between folic acid supplementation and the incidence of melanoma was inferred using the IVW method (OR = 0.88, 95% CI 0.80–0.96, p = 0.006) and the weighted mode, simple median and MR-Egger, dramatically aligned in their findings. The Cochran Q-test for heterogeneity, utilized in the IVW method, revealed no significant heterogeneity in our study (P = 0.844). The non-significant result of the Cochran Q-test enhances the study's validity by showcasing the homogeneity of the genetic instruments employed [43]. Our risk estimates remained uninfluenced by directional pleiotropy, evidenced by the near-null average pleiotropic effect (MR-Egger intercept < 0.001, p = 0.905). Despite the seemingly small decrease in risk, this finding has important implications for public health due to the severity and growing prevalence of melanoma [44].

**Table 2:** Detailed SNP related to folic acid supplementation findings of the causal association between folic acid supplementation and melanoma incidence

Gene	SNP	Effect allele	Other allele	Beta	SE	P value	F value
HLA-DQA2	rs6605556	G	A	0.004	< 0.001	1.69e-52	232.444
HLA-DQA1	rs185774696	T	C	0.001	< 0.001	2.5e-06	22.179
HLA-DQA1	rs529480034	T	C	0.002	< 0. 001	1.69e-18	77.019
PHTF1	rs6679677	A	C	0.002	< 0. 001	1.39e-11	45.670
THBS2	rs6903479	C	T	0.001	< 0. 001	4.30e-06	21.124
CSMD1	rs10089891	T	C	-0.001	< 0. 001	2.5e-06	22.129
ATXN2	rs597808	G	A	-0.001	< 0. 001	2.99e-07	26.228
None	rs2047847	C	A	0.001	< 0. 001	4.49e-06	21.038
None	rs6910879	G	A	0.004	< 0. 001	1.80e-24	104.227
None	rs35175534	C	A	0.004	< 0. 001	2.60e-33	144.592

### Robustness of the causal relationship between folate supplementation and melanoma

In a crucial "leave-one-out" sensitivity analysis for robustness, IVW estimates after excluding each SNP closely mirrored those from the full set, suggesting no single SNP significantly influenced the causal relationship (see supplementary Fig.). This analysis prevents a single SNP from skewing the effect size excessively, highlighting IVW's robustness against inflated or misleading inferences. Minimal deviation in revised estimates indicates no SNP disproportionately affects results, reinforcing the genuine correlation found across genetic variation.

### LDSC regression analysis

We conducted LDSC regression analysis to assess the genetic correlation between folic acid supplementation and melanoma. The results, presented in Table 3, showed a weak genetic correlation (rg = 0.199, p = 0.805).

**Table 3:** The genetic correlations between folate supplementation and Melanoma

Trait 1	Trait 2	r_g_	SE	p-Value
Folic acid supplementation	Melanoma	0.199	0.806	0.805

### MRlap analysis

Table 4 shows the results of the MRlap analysis. The significant difference in p-values before and after correction indicates that sample overlap affects our conclusions. However, with a p-value difference of 0.136, the overall impact of sample overlap on the results is not significant.

**Table 4:** Results of MRlap analysis

Exposure	Outcome	Observed effect p-value	Corrected_effect SE	Corrected effect	Corrected effect p-value	p-difference
Folic acid supplementation	Melanoma	0.002	1.673	2.377	0.155	0.136

### Meta-analysis

**Fig. 4:**
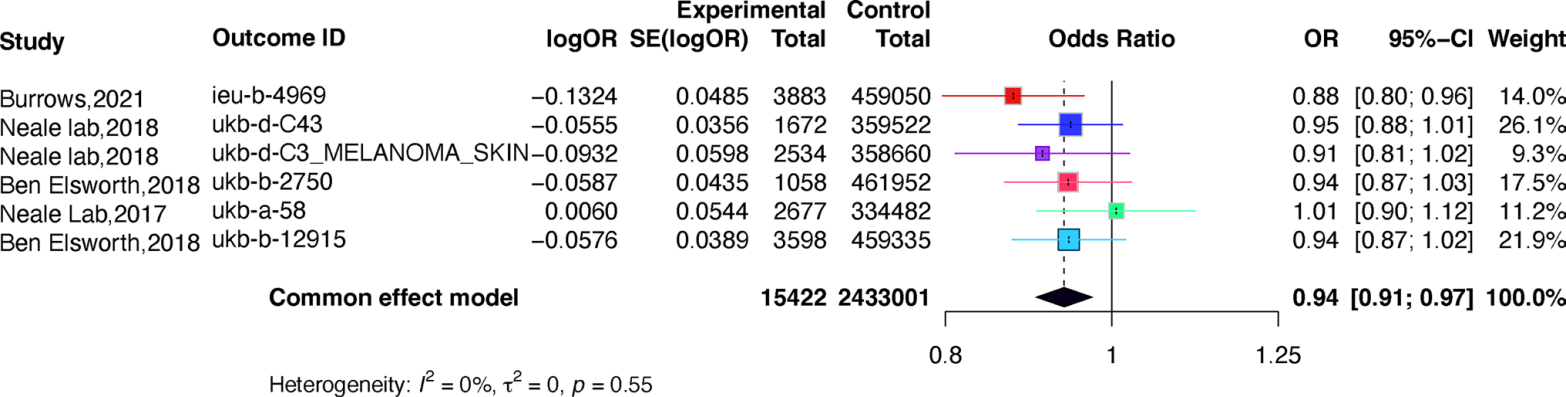
Forest plot of studies that evaluated the causal effect between folate supplementation and melanoma outcomes using values obtained by the IVW MR method

**Fig. 5:**
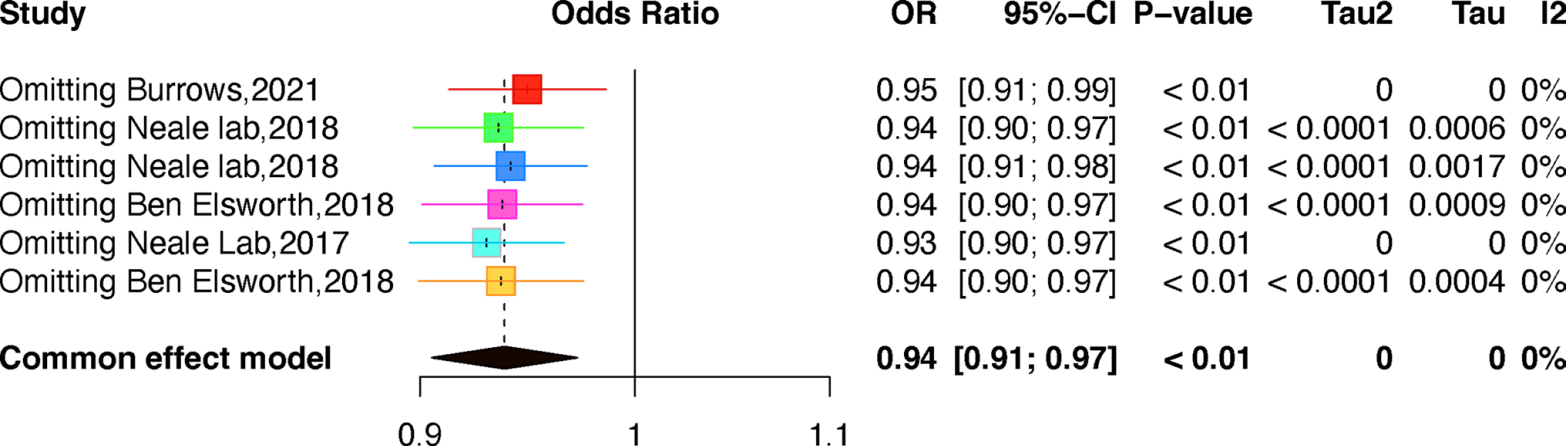
The influence of each study on the outcome of the meta-analysis between folate supplementation and melanoma

Meta-analysis of the IVW method-derived MR estimates for melanoma (Fig. 4). The direction and magnitude of the association were consistent in 5 GWAS datasets. Sensitivity analysis was performed by reducing one outcome at a time to evaluate the impact of a single study on this meta-analysis. Figure 5 shows the results of the pooled calculated after deleting one of the outcomes. As can be seen from the table, the combined ORs after elimination have not changed significantly (P < 0.01), indicating that the results of this analysis are not overly dependent on a certain study and the conclusion is stable. The sensitivity analysis results of the above indicators indicate the stability of the results of the causal effect study of folate supplementation on melanoma.

## Discussion

Our study found a negative causal relationship between folate supplementation and melanoma. The evidence on the link between folate and overall/site-specific cancer risk is inconsistent, and there are limited epidemiological studies. For example, a French study that followed a cohort for 20 years revealed a higher risk of overall skin cancers associated with dietary folate intake [45, 46]. Despite mandatory folic acid fortification for pregnant women in the United States and Canada, many European, Asian, and African nations have yet to enforce such fortification. One significant concern among adults is the potential elevated cancer risk associated with folate consumption [47]. Our research using a two-sample Mendelian randomization (MR) approach has revealed a causal link between folate supplementation and a reduced risk of cutaneous melanoma (CM), indicating the pharmacological effects of folate product supplementation might contribute to the decline in skin cancers occurrences. To our knowledge, this is the initial study to explore the potential causal links of folate supplementation with melanoma using genetic data, although specific folate dosages and frequency are not mentioned in GWAS data. In recent years, extensive research has focused on the impact of vitamin B6 on cancer. Mikkelsen's [48] study investigated the immune response modulation effects of B vitamins and found that vitamin B2 (riboflavin), B6 (pyridoxine), and B9 (folic acid) displayed antitumor activity in promonocytic lymphoma cell lines. These findings could pave the way for future investigations into using vitamin B supplementation to curb cancer cell growth in vivo. Possible mechanisms for the anti-proliferative and anti-migratory effects of these vitamins include angiogenesis, altered cytokine secretion, modified PD-L1 expression (as a programmed cell death ligand), oxidative stress, and nitric oxide synthesis. A review by Mocellin [49] highlights an inverse linear relationship between cancer risk and both dietary intake of vitamin B6 and PLP levels, with the strongest link observed in gastrointestinal cancer. Both studies pose the same question: whether these vitamins directly prevent cancer, or they are merely markers in healthy individuals' plasma and indicators of vitamin-rich diets. The conclusions remain unclear, necessitating further research into both hypotheses.

Folic acid has a well-established role in DNA synthesis and repair. Ames [50] suggests that folate deficiency, along with potential deficiencies in vitamin B12 and B_6_, are linked to cancer due to the insertion of uracil instead of the correct base into human DNA, leading to chromosomal breaks. A study [51] involving Hawaiian women indicated that B vitamin may protect against cervical cancer by reducing premalignant cervical lesions with high nutrient intakes [52]. There are indications of interactions between micronutrient intake levels, genotype, and cancer risk. Different forms of the methylenetetrahydrofolate reductase (MTHFR) gene carry varying risks of colon cancer. The CC genotype, along with low micronutrient intake, poses the highest risk of colon cancer [53, 54]. Nutritional genomics is a relatively new field, and further research is needed to validate any observed interactions. Additionally, specific folic acid-related SNPs might influence melanoma risk biologically. THBS2 (thrombospondin-2) is implicated in the regulation of melanoma progression, potentially influencing tumor growth and metastasis [55]. Depending on an individual's genetic background, purity of the HLA-DQA1 locus may be considered a potential risk factor for the development of melanoma, having the ability to suppress melanoma cell growth, proliferation, and migration [56, 57]. Other research found that class II molecules (HLA-DPA1, HLA-DPB1, HLA-DQA1, HLA-DRA, HLA-DRB1, HLA-DRB5) are important biomarkers in the occurrence and progression of melanoma tumors, and their expression levels are closely related to prognosis and immune infiltration [58]. CSMD (CUB and Sushi Multiple Domains 1) can interact with Smad3, confirming the role of CSDM1 as a tumor suppressor gene in melanoma cells [59]. These findings are consistent with the majority, though not all, of previous studies conducted on this topic.

Meta-analysis of machine-controlled trials shows no significant effect of folic acid supplementation on the risk of melanoma skin cancers [60], while another meta-analysis of three trials found an inverse association between folic acid supplements and melanoma risk [61]. Our standpoint received additional backing from a subsequent analysis. There are currently no published prospective cohort analyses specifically examining the relationship between dietary folate/folic acid and melanoma. The utilization of folate supplementation in cancer is intricate but warrants attention, especially considering many cancers have an epigenetic foundation. Compared to previous findings, our Mendelian randomization (MR) results are more robust as they mitigate confounding factors and causal association biases to some extent. Consequently, we observed a significant association between folate and melanoma.

However, our study has certain limitations. Firstly, the validity of the MR study might be compromised due to weak instrumental variables (IVs). To address the validity of MR study, we utilized independent single nucleotide polymorphisms (SNPs) reaching genome-wide significance levels (P < 5 × 10–6), and the F-statistic met the threshold of > 10 to avoid the effect of weak instruments. Nevertheless, it is still necessary to conduct sensitivity analyses to assess the potential influence of weak instruments and improve the robustness of the causal inference. Secondly, Pleiotropy occurs when genetic variants influence multiple traits, beyond the exposure of interest. While several statistical approaches were employed to detect and adjust for pleiotropic effects, these methods have their own limitations. As such, the potential for residual pleiotropy cannot be fully excluded. A larger GWAS sample size is necessary to identify more folate-related SNPs in the future. Also, pleiotropy could arise when some instrumental SNPs are linked to multiple other factors. To our knowledge, this is the first study exploring the potential causal associations of folic acid supplementation with melanoma based on genetic data yet there is no mention of folate-specific dosage in GWAS data. Additionally, since our study's subjects are limited to Europeans, the results may not generalize to the entire population, and any research conclusions should be cautiously extrapolated. In addition, since our study's subjects are limited to Europeans, the results may not generalize to the entire population, and any research conclusions should be cautiously extrapolated. Genetic heterogeneity across populations may lead to differences in allele frequencies and the biological mechanisms underlying the traits studied. As such, future studies should aim to include data from more diverse ethnic groups to ensure that findings are broadly applicable. This will help enhance the external validity of Mendelian randomization studies and ensure that the identified causal relationships are more representative. Nevertheless, while the association between folate and melanoma risk is statistically significant, it is vital to clarify that the causal relationship inferred from the data requires further validation. As heterogeneity across ethnicities, genetic backgrounds, and environmental contexts could influence the generalizability of the results. In addition to the biological mechanisms, potential confounding factors must be carefully considered to avoid overstating the association. Lifestyle factors such as sun exposure, diet, alcohol consumption, and smoking have been implicated in both folate metabolism and melanoma risk. Failure to account for these confounders may lead to biased conclusions, obscuring the true nature of the relationship between folate and melanoma. Meanwhile, we acknowledged the need for further research to definitively establish this link. All in all, MR analysis can only establish causal relationships between exposure and skin cancers but cannot provide the specific effect sizes of exposure on skin cancers. We used GWAS data for analysis and performed meta-analysis, but these data came from public databases, and there were problems such as small data size and confounding factors. Therefore, further experiments or trials are needed to validate these results.

## Conclusion

In summary, genetically determined folate supplementation was associated with a decreased risk of melanoma using MR methods and meta-analysis. Our research suggests that folate supplementation may be a potential preventive measure for melanoma, which provides a rationale for using folate supplementation as a promising target for melanoma prevention. Specifically, we recommend that individuals at a higher risk of melanoma due to genetic predisposition, long-term UV exposure, or other established risk factors may consider folate supplementation. For the general population, more research is needed to assess the overall benefits and risks of folate supplementation to develop more personalized prevention strategies. Further studies are needed to determine how folate affects the occurrence and development of melanoma and confirm the association in large-scale randomized controlled trials and scientific animal experiments.

## Supplementary Information

Additional file 1.

Additional file 2.

Additional file 3.

## Data Availability

The original contributions presented in the study are included in the article/Supplementary Material. Further inquiries can be directed to the corresponding authors.

## Abbreviations

LDSC: Linkage disequilibrium score regression
MR: Mendelian randomization
SNPs: Single-nucleotide polymorphisms
IVW: Inverse-variance weighted
OR: Odds ratio
CI: Confidence interval
STROBE-MR: Strengthening the reporting of observational studies in epidemiology for mendelian randomization
BCC: Basal cell carcinoma
SCC: Squamous cell carcinoma
CM: Cutaneous melanoma
NMSC: Non-melanoma skin cancers
GWAS: Genome-wide association studies
IVs: Instrumental variables
UK Biobank: A large-scale biomedical database and research resource
EBI: European bioinformatics institute
MAF: Minor allele frequency
P-value: Probability value
LD: Linkage disequilibrium
MRlap: Mendelian randomization with overlapping samples
PLP: Pyridoxal 5'-phosphate (active form of vitamin B6)
MTHFR: Methylenetetrahydrofolate reductase
HLA-DQA1: Human leukocyte antigen DQA1
