# Supplementary material for: Genetically predicted vitamins supplementation and risk of skin cancers: a Mendelian randomization study
Source: Discov Oncol. 2025 Feb 19;16:204. doi: 10.1007/s12672-025-01905-9 (PMC11839550; doi:10.1007/s12672-025-01905-9)

Forest plot to visualize Mendelian randomization estimates from instrument variants for folate acid supplementation on risk of melanoma by three methods. IVW, inverse-variance weighted; MR, Mendelian randomization; OR, odds ratio.


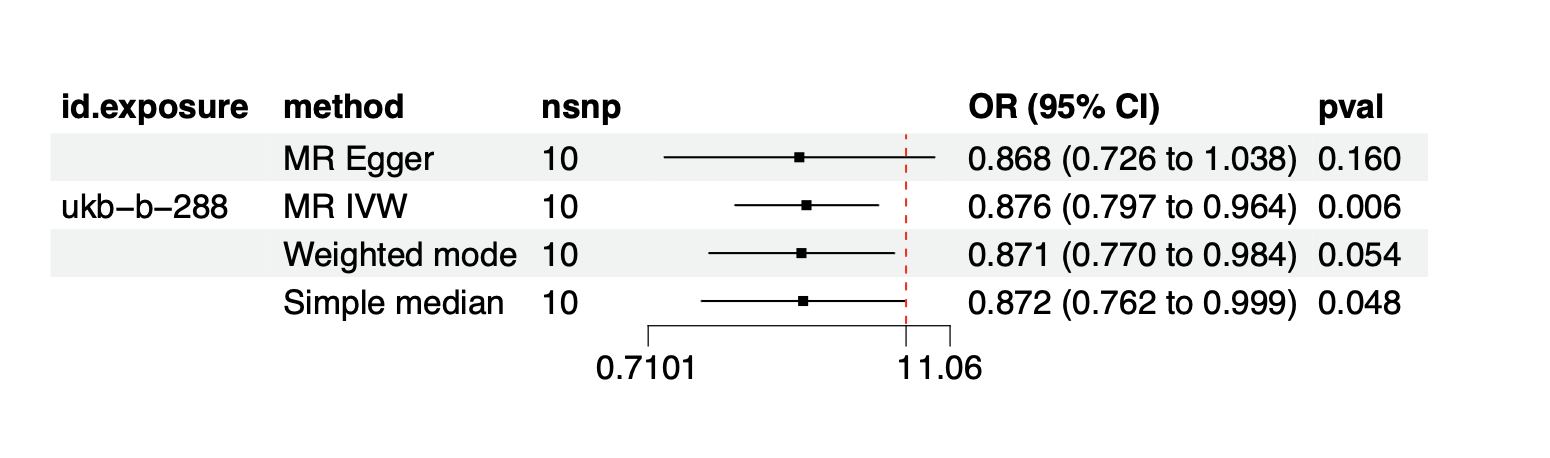


Funnel plot of the estimated causal effect of folic acid supplementation on melanoma skin cancer incidence. Each point symbolizes the estimated causal effect of each instrumental variable (IV). The dark blue vertical line represents the MR-Egger method-derived causal effect estimate, while the light blue line signiﬁes the equivalent estimate derived via the IVW method.


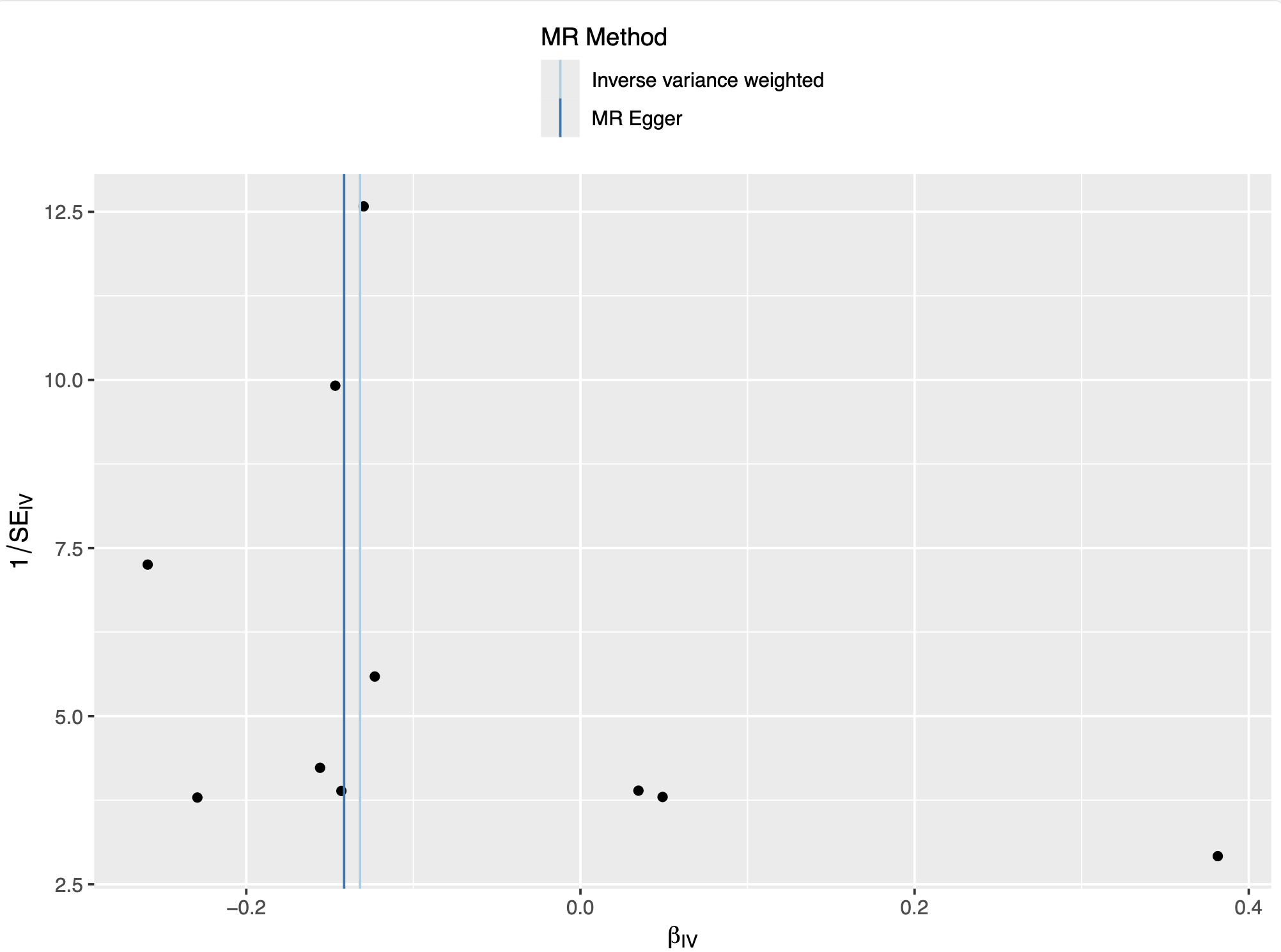


Leave-one-out plot to visualize causal effect of folic acid supplementation the risk of cutaneous melanoma when leaving one single-nucleotide polymorphism (SNP) out.


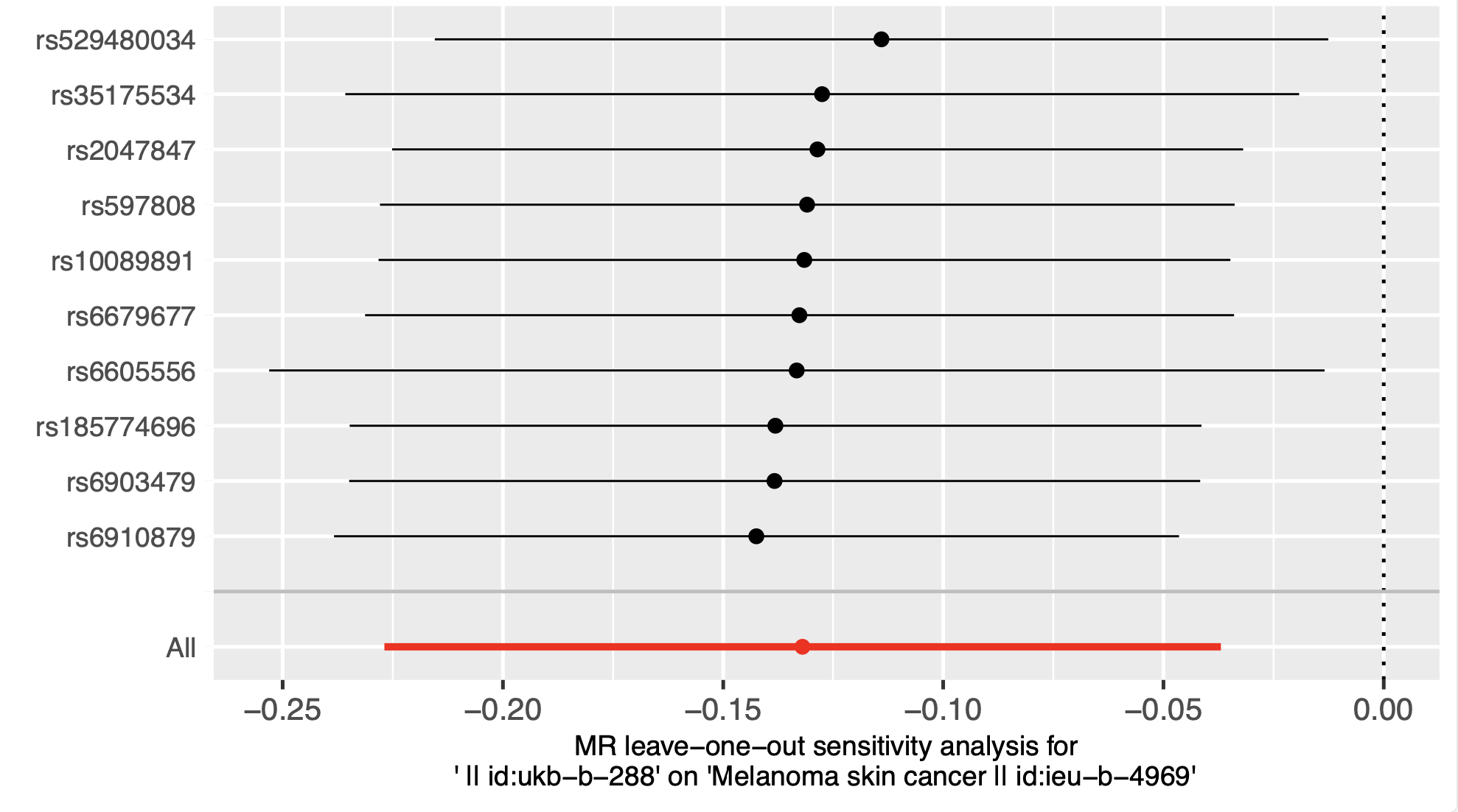


Scatter plot illustrating the associations between melanoma skin cancer (y-axis) and folic acid supplementation(x-axis). The slope of the straight line indicates the magnitude of the causal association. The light blue line represents MR-Egger regression, the light green line represents weighted median, and the dark blue line represents inverse-variance weighted (IVW). SNP, single-nucleotide polymorphism.


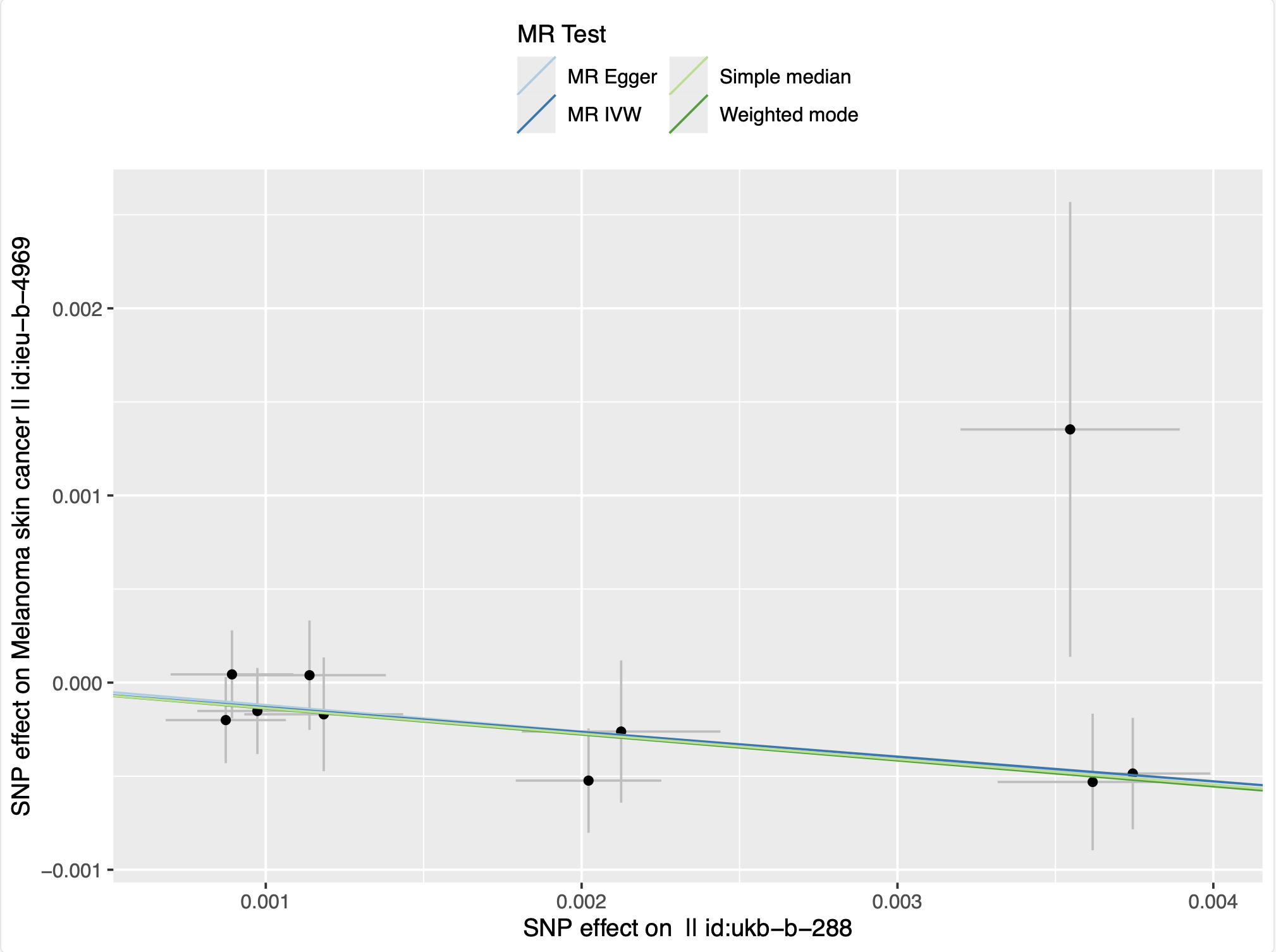

Supplement: Supplementary file 3 — Additional file 3. [file 12672_2025_1905_MOESM3_ESM.docx]
